# Developing an AI-powered tool for radiographic feedback on working length determination in pre-clinical endodontic training

**DOI:** 10.3389/fdmed.2026.1730454

**Published:** 2026-02-27

**Authors:** Sanaa Aljamani, Iman AlMomani, Walid El-Shafai, Mousa AL-Akhras, AbdulAziz AlHaddad, Rawan Abu zaghlan

**Affiliations:** 1 Restorative Department, School of Dentistry, The University of Jordan, Amman, Jordan; 2 Consultant in Endodontics, Restorative Department, Jordan University Hospital, Amman, Jordan; 3 Computer Science Department, King Abdullah II School of Information Technology, The University of Jordan, Amman, Jordan; 4 Intelligent Cybersecurity Engineering Research Group, The University of Jordan, Amman, Jordan; 5 Computer Science Department, CCIS, Prince Sultan University, Riyadh, Saudi Arabia; 6 Department of Electronics and Electrical Communications Engineering, Faculty of Electronic Engineering, Menoufia University, Menouf, Egypt; 7 Automated Systems and Computing Lab (ASCL), Prince Sultan University, Riyadh, Saudi Arabia; 8 Computer Information Systems Department, King Abdullah II School of Information Technology, The University of Jordan, Amman, Jordan

**Keywords:** artificial intelligence, constructive feedback, convolutional neural network (CNN), education technology, endodontic education, ground-truthing, machine learning, radiographic

## Abstract

**Background:**

Establishing an accurate working length is a critical step in root canal treatment and directly influences clinical success. As artificial intelligence increasingly integrates into medical education, applying it to enhance endodontic training has become increasingly important.

**Aims:**

This study aimed to develop a machine learning–based tool that provides prompt, personalized, constructive feedback on radiographic working length determination in a pre-clinical setting and to evaluate its usability among dental students.

**Methods:**

A newly labeled dataset of 3,000 radiographic images was created and categorized into optimal, over-extended, and under-extended working lengths. This dataset was balanced and split into 80%, 10%, and 10% for training, validation, and testing, respectively. Twenty-two convolutional neural network models were developed, trained, and evaluated using five diagnostic metrics (accuracy, F1-score, precision, recall, and testing time). The best-performing model was integrated into a web-based platform and piloted with 30 pre-clinical dental students who provided usability feedback via a Likert-scale questionnaire. The study hypothesized that students would rate the tool as usable and educationally supportive.

**Results:**

The custom-developed deep CNN achieved 97%–99% accuracy, 95%–98% F1-score, 94%–99% precision, and a recall rate of 96%–98%, with an average testing time of 0.54 s. Students rated the proposed system positively across clarity, ease of use, and learning support, with median usability scores of 5.0 across all items and interquartile ranges of 4–5 to 5–5.

**Conclusion:**

The AI-powered feedback system demonstrated high accuracy with strong user acceptance. By delivering instant, constructive feedback on working length determination, it supports effective learning and skill refinement in endodontic education. It is also beneficial in classrooms with large student populations. Future work will expand the dataset and integrate additional stages of root canal training into a unified AI-based educational platform.

## 1 Introduction

**Figure 1:**
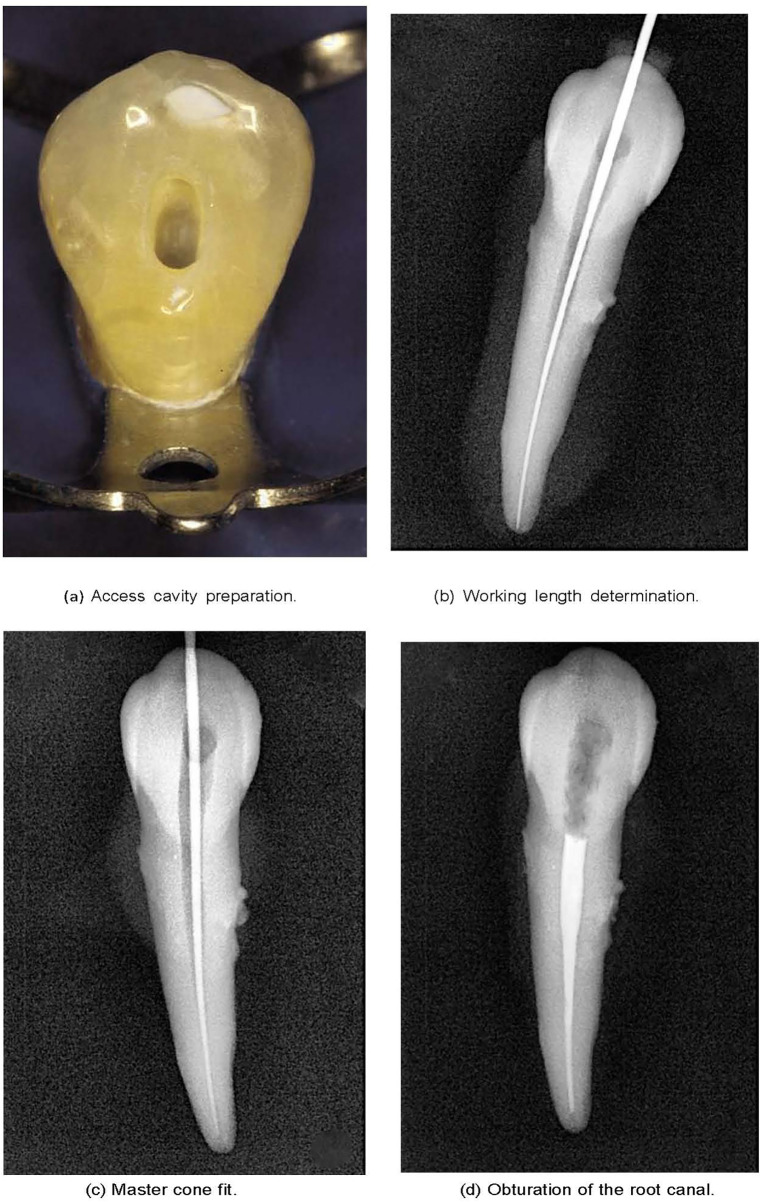
The technical stages of root canal treatment.

Root canal treatment (RCT) is a fundamental procedure in endodontics aimed at eliminating dental pulp infections through the cleaning and shaping of the root canal system, utilizing specialized instruments and materials (1, 2). Figure 1 illustrates different stages of RCT, which involve chemical and mechanical cleaning to the correct working length of the root canal and filling the space with a dedicated root canal-filling material.

The success of RCT is influenced by various factors, including tooth-related aspects, accurate diagnosis, and careful interpretation of radiographic examinations (3, 4). The technical stages of RCT follow a sequential approach, beginning with access cavity preparation, followed by working length (WL) determination, chemo-mechanical preparation, and obturation. Each step is codependent, with the accuracy of one step directly affecting the next (1, 4, 5).

Working length (WL) in RCT defines the extent to which cleaning and shaping should be performed within the root canal (4). Literature suggests that WL should terminate within 2 mm of the radiographic apex to optimize treatment outcomes (6–8). Incorrect working length determination can lead to undesirable technical and biological consequences of the whole endodontic procedure (6, 9, 10). Historically, WL has been determined using various techniques, including patient response and tactile sensation. However, these techniques are often inaccurate due to factors such as root canal morphology, apical inflammation, and patient variability (11, 12). Electronic apex locators’ technique has demonstrated improved accuracy in WL determination and reduced radiation exposure, but is also associated with limitations when used alone (8, 13, 14). A combined approach incorporating radiographic WL measurements is recommended to ensure reliable results of WL determination (7, 15, 16).

Artificial intelligence (AI) is increasingly being adopted in the dental field to enhance efficiency and accuracy (17–20). AI-driven machine learning models based on radiographic and image analysis have shown promising results in diagnosing dental caries (21), detecting periapical pathology and diagnosis (19, 22–24), assessing the quality of root canal filling (25, 26), and improving orthodontic treatment planning (27, 28).

The research on AI applications in WL determination remains limited (28). In one study, AI-driven models for WL determination have demonstrated considerable accuracy, with some models achieving 96% accuracy compared to specialist endodontists 76% (29). Another model achieved 85% accuracy compared to the dual-frequency impedance ratio method (30). Table 1 summarizes relevant studies that explored AI work in radiographic examination in endodontics.

**Table 1:** Summary of AI methods used in dental radiography studies.

Author and year	Diagnostic technique	AI method	Outcome measure
Saghiri et al., 2012	Periapical radiographs/cadaver study	ANN alone	93% accuracy in working length determination
Saghiri et al., 2012	Periapical radiographs/adaver study	ANN compared to Endodontists	96% accuracy in anatomical position of minor apical foramen compared to 76% of endodontists
Qiao et al., 2020	Circuit system designed for working length measurements	Neural network-based multi-frequency impedance method	95% accuracy in working length determination
HA et al., 2023	Radiographs/ Augmented images	Deep learning Model (YOLOv5s, YOLOv5x, and YOLOv7)	Denoising and data balancing of radiographic images have set the accuracy of the three models to be above 95%
Michael G et al., 2020	Panoramic radiographs	Predictive deep learning algorithm	The rank correlation between model and cohort confidence scores for positive and negative condition cases was 0.72 and 0.34, respectively.
X Gao et al., 2021	Radiographic and clinical data	BP artificial neural network model	The accuracy of this BP neural network model was 95.60% for the prediction of post-operative pain.
NP M et al., 2022	Periapical radiograph (3000)	CNN to score periapical lesion on periapical radiographs using the PAI score system	True prediction PAI 1: 90.0%, PAI 2,5: 30%, PAI 3: 60%, PAI 4: 71%
MJ et al., 2017	Periapical radiograph and CBCT (*N* = 240)	Probabilistic neural network (PNN)	PNN in CBCT images was 96.6% accurate in detecting vertical root fracture

Undergraduate dental education introduces students to the science and practice of endodontics through pre-clinical training, where they develop knowledge, skills, and confidence via theoretical and practical sessions (31–34). These sessions emphasize the importance of key factors influencing successful RCT, such as understanding tooth anatomy, accurate diagnosis, careful interpretation of radiographs, and practical steps of root canal treatment (3, 35, 36).

While AI integration in dental education has been generally well-received, such as virtual reality simulators for operative dentistry (37–39) and AI-assisted diagnostic assessments in endodontics (40), existing WL-related AI studies do not provide personalized, immediate, or formative feedback, which is essential for skill acquisition in a pre-clinical setting.

The precise gap addressed in this study is the absence of an AI-based educational tool that moves beyond simple image classification to deliver instant, individualized feedback on radiographic working length determination. Instant formative feedback enables students to recognize errors, self-correct, and refine their technique during the learning process; an advantage not offered by previous WL-focused AI models, which provide only categorical predictions without pedagogical guidance.

This study therefore aimed to develop machine learning models to provide targeted feedback on radiographic working length (WL) determination through the following steps:

Create a new, balanced, labeled dataset of single-rooted tooth radiographs categorized into optimal, over-extended, and under-extended WL.
Train and test 22 convolutional neural network (CNN) models on this dataset.
Evaluate models’ performance using 11 quantitative metrics.
Select the highest-performing model—primarily based on accuracy—to develop an innovative, automated, and instant web-based WL feedback system.
Pilot the system with pre-clinical dental students to assess its usability and educational effectiveness.

## 2 Material and methods

### 2.1 Proposed AI-driven framework

**Figure 2:**
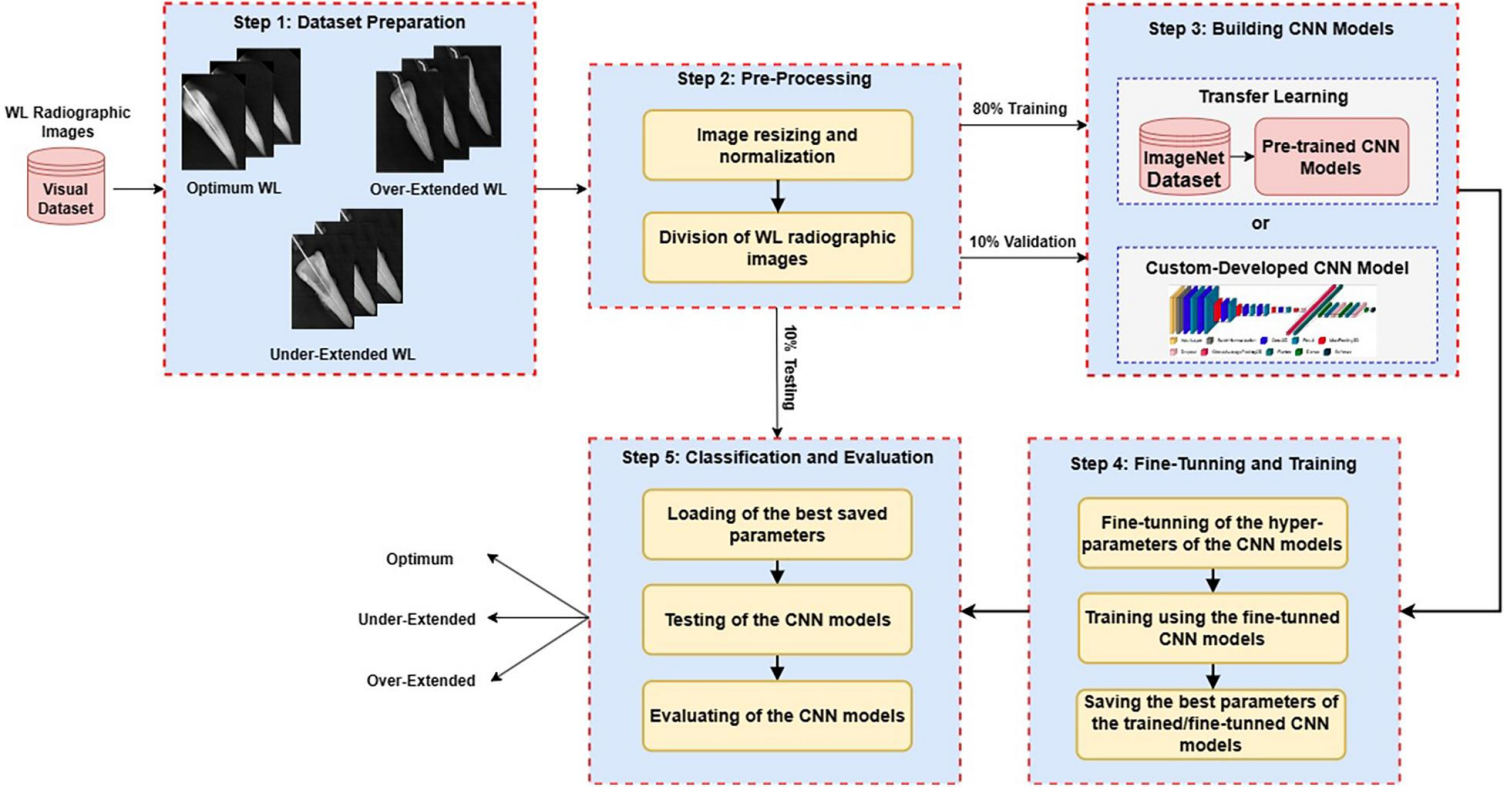
The main steps of the preposed AI-driven framework.

The methodology in this study outlines a detailed and systematic approach to developing a highly accurate AI-based system for working length determination using dental radiographic images in dental education. This proposed AI-driven framework incorporated both pre-trained and custom-developed Convolutional Neural Network (CNN) models, utilizing their capabilities to overcome the complexities inherent in dental radiographic images. Figure 2 presents a schematic overview of the primary components encompassed within the proposed framework. Subsequent subsections detail the various phases in this framework, providing a comprehensive roadmap of the methodological approach adopted by the IT team in this study.

An AI-assisted feedback tool was developed and integrated within a web-based platform to provide automated evaluation and feedback on students’ laboratory performance. The system was piloted with a group of 30 dental students (≈10% of the enrolled cohort of 300 students) who voluntarily agreed to participate towards the end of their pre-clinical year. This is justified as an appropriate pilot sample size, as 10–30 participants are considered sufficient to identify usability patterns and system acceptability.

After using the tool during their laboratory sessions, participants completed a structured feedback questionnaire to evaluate its effectiveness and usefulness. The questionnaire employed a five-point Likert scale (1 = strongly disagree to 5 = strongly agree) covering domains of usability, reliability, feedback clarity, and overall satisfaction.

### 2.2 Dataset preparation

The foundational step of the proposed framework is to assemble a robust dataset. This dataset initially consists of 321 high-resolution digital radiographs and has been expanded to include 3,000 digital radiographs of anterior and premolar human teeth, accessed and extracted using K files of minimum size 15, to measure the WL. These images were obtained from pre-clinical laboratory simulation exercises using extracted teeth only; no patient radiographs were used. Ethical approval for the use of extracted human teeth and radiographic imaging for research and educational purposes was obtained from the Institutional Review Board (IRB), University of Jordan (Decision No. 547/2025). All radiographs were fully de-identified prior to analysis in accordance with IRB-approved procedures and institutional research governance requirements.

The WL dental radiographic images were collected and obtained using the advanced Ai Dental Woodpecker-V1.0.20 imaging software with acquisition parameters of (65 kVp, 7 mA, and an exposure time of 0.17 s).

Duplicate images were identified and removed to maintain data uniqueness and quality, ensuring dataset integrity, robustness, and utility. Concurrently, an extensive labeling operation was conducted, in which each image was systematically annotated to categorize the working length as optimal, under-extended, or over-extended by both a specialist endodontist and a radiologist with 4–5 years of experience. Both annotators were calibrated using 50 sample images prior to labeling the entire dataset. Inter-rater agreement was quantified using Cohen's *κ*, yielding a *κ* value of 0.77 (95% CI: 0.72–0.82), indicating substantial agreement. Disagreements were resolved through adjudication by a third senior endodontist.

The categorization protocol, developed specifically for this study, relied on precise measurements of the distance between the endodontic file tip and the radiographic root apex as recommended in the literature (4, 6, 7). The collected images were almost evenly distributed across the classifications: 1,007 radiographic images with optimum WL, 1,001 with under-extended WL, and 1,004 with over-extended WL.

Per-class counts were maintained across training, validation, and test sets using a stratified split. The final partition included 80% for training (*n* = 2,400), 10% for validation (*n* = 300), and 10% for test (*n* = 300). Splits were performed at the tooth level to prevent data leakage, ensuring no radiographs of the same tooth appeared across different subsets. Near-duplicates were removed prior to splitting. No class-specific augmentation was applied beyond standard transformations used uniformly across all categories.

**Figure 3:**
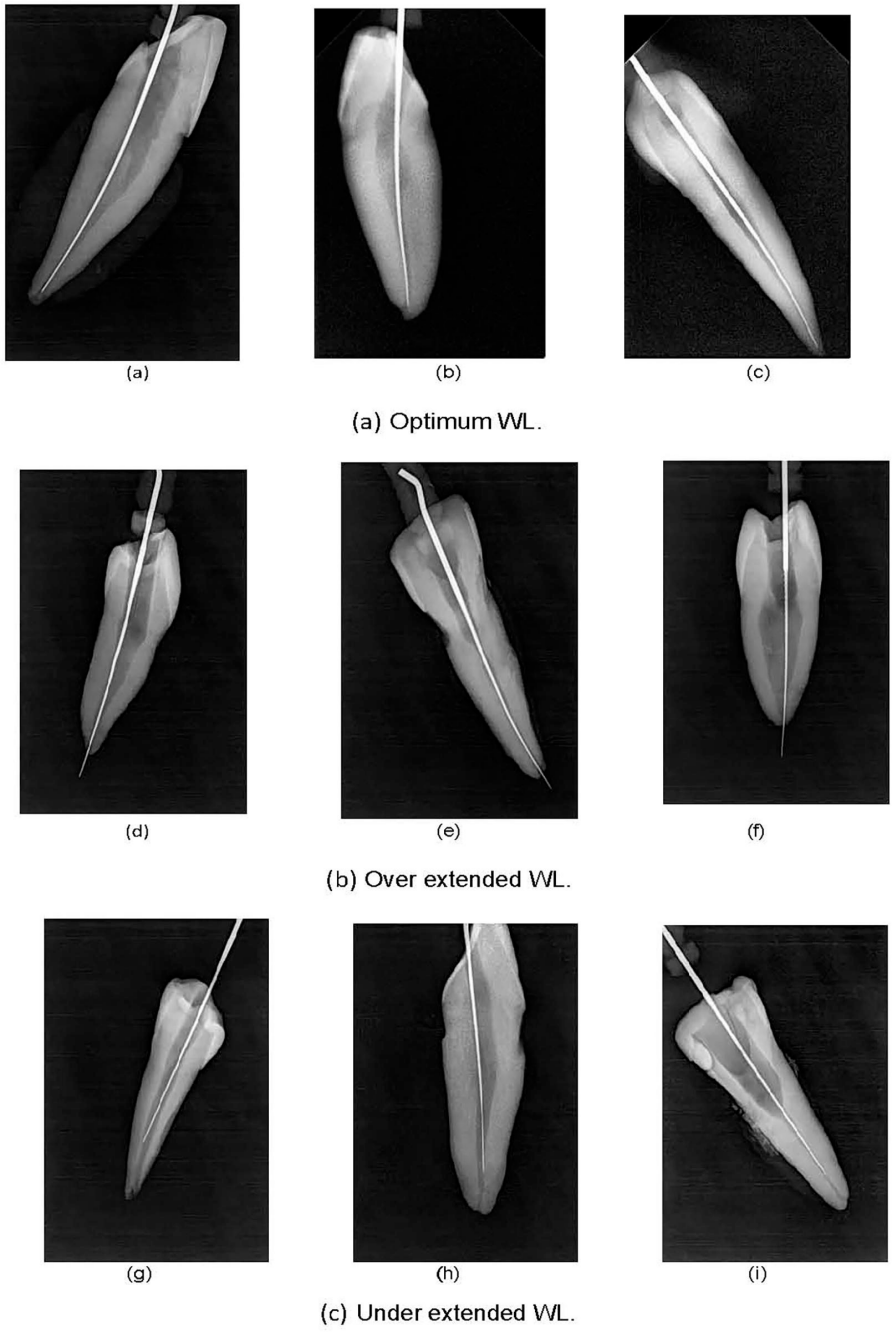
Visual samples of various examined working lengths: **(a)** Optimum WL, **(b)** over extended WL, and **(c)** under extended WL.

Samples of WL radiographs, categorized according to the classification process, are presented in the representative images in Figure 3, with each numerical category described as follows:

**0**: Optimum WL images, where the file tip is optimally positioned within a 0–2 mm range from the radiographic apex, ensuring that the cleaning and shaping of the root canal system is within the confines of the root structure.
**1**: Under-extended WL images, where the file tip falls short of reaching the radiographic apex by more than 2 mm, potentially leading to inadequate disinfection.
**2**: Over-extended WL images, where the file tip extends beyond the radiographic apex, an undesirable outcome risking over-instrumentation of the root canal system.

All WL digital radiographic images were resized to a uniform resolution of 224 × 224 pixels. Resizing helps reduce computational load and streamline the processing pipeline (41, 42).

Because the dataset consisted exclusively of single-rooted anterior and premolar teeth, generalizability to multi-rooted molars, anatomical variations, and different radiographic sensors may be limited. This limitation is addressed further in the discussion section. No external validation dataset from another clinic or imaging system was available for this study.

### 2.3 Building CNN models

This study employed a dual-pathway model development strategy which encompasses both the integration of state-of-the-art pre-trained CNN architectures [VGG16, ResNet50, VGG19, DenseNet121, DenseNet169, DenseNet201, EfficientNet series (EfficientNetB0- EfficientNetB7), InceptionResNetV2, InceptionV3, MobileNet, MobileNetV2, Mo- bileNetV3Large, MobileNetV3Small, and Xception] (43–48), and the development of a custom CNN model designed to capture the unique features of endodontic radiographic imaging that generic and pre-trained models may not effectively capture. This aims to harness the robust capabilities of advanced neural networks while addressing the specific challenges associated with dental radiographic images.

A diverse selection of pre-trained CNN architectures was integrated, each offering distinct advantages in image recognition. VGG16 and VGG19 captured texture and details, while ResNet50 and DenseNet series addressed deep network training challenges. EfficientNet models optimized accuracy with minimal computational cost, and InceptionResNetV2 and InceptionV3 improved learning efficiency. MobileNet variants and Xception achieve a balance between speed and accuracy, making them ideal for resource-limited applications.

We began by integrating and selecting a diverse array of proven pre-trained CNN architectures, each known for its distinct advantages in various aspects of image recognition. These architectures include:

**VGG16 and VGG19**: Renowned for their simplicity and deep architecture, which is particularly effective for capturing image texture and details.
**ResNet50 and DenseNet Series** (**DenseNet121, DenseNet169, DenseNet201**): These models leverage residual and dense connections, respectively, to facilitate training deeper networks by alleviating the vanishing gradient problem.
**EfficientNet Series** (**EfficientNetB0**—**EfficientNetB7**): Known for scaling up CNNs in a more structured manner to achieve higher accuracy without high computational costs.
**InceptionResNetV2** and **InceptionV3**: These models combine inception modules with residual connections to improve learning speed and accuracy.
**MobileNet Series** (**MobileNet, MobileNetV2, MobileNetV3 Large, MobileNetV3 Small**) and **Xception**: Optimized for mobile devices, these models offer a good balance between speed and accuracy, making them suitable for applications where computational resources are limited.

Each of these pre-trained models is fine-tuned to adapt to our dental imaging dataset. This involves modifying the top layers of the network to focus on features specific to dental radiographs, such as tooth anatomy and the positioning of endodontic files.

**Figure 4:**
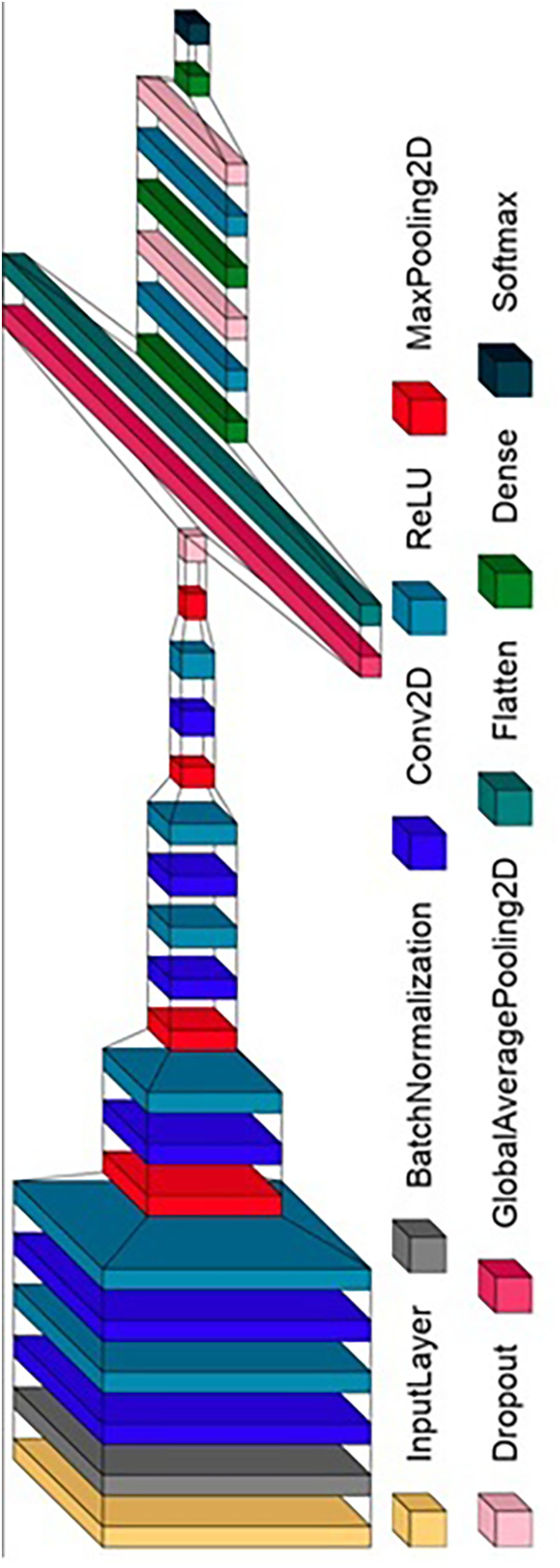
Detailed architecture of the custom-developed CNN model.

To complement the broad learning capabilities of the pre-trained networks, we also developed a custom CNN architecture tailored to the nuanced requirements of endodontic radiographic analysis. The architecture of this custom model is carefully designed with several specialized layers, as demonstrated in Figure 4; Table 2:

**Convolutional Layers**: Configured to detect fine-grained details critical for accurate working length determination.
**Depthwise Separable Convolutions**: Implemented to provide efficient model scaling and detailed feature extraction without a significant increase in computational demand.
**Dilated Convolutions**: Used to expand the receptive field of the network, allowing it to encompass broader contextual information without losing resolution.

**Table 2:** Layer-by-layer architecture of the custom-developed CNN model.

Type of the layer	Output configuration	Parameter characteristics
Batch Normalization (batch normalization)	224 × 224 spatial resolution, 3 channels	Count: 12, Normalization parameters
2D Convolution (conv2d)	224 × 224 spatial resolution, 8 units	Count: 224, Feature detectors
2D Convolution (conv2d 1)	224 × 224 spatial resolution, 16 units	Count: 1,168, Feature detectors
Max Pooling (max pooling2d)	Halved resolution: 112 × 112, 16 units	None, Dimensionality reduction
2D Convolution (conv2d 2)	Halved resolution: 112 × 112, 32 units	Count: 4,640, Feature detectors
Max Pooling (max pooling2d 1)	Quartered resolution: 56 × 56, 32 units	None, Dimensionality reduction
2D Convolution (conv2d 3)	Quartered resolution: 56 × 56, 64 units	Count: 18,496, Feature detectors
2D Convolution (conv2d 4)	Quartered resolution: 56 × 56, 64 units	Count: 36,928, Feature detectors
Max Pooling (max pooling2d 2)	One-eighth resolution: 28 × 28, 64 units	None, Dimensionality reduction
2D Convolution (conv2d 5)	One-eighth resolution: 28 × 28, 256 units	Count: 147,712, Advanced feature extraction
Max Pooling (max pooling2d 3)	One-sixteenth resolution: 14 × 14, 256 units	None, Dimensionality reduction
Dropout (dropout)	Preserved resolution: 14 × 14, 256 units	None, Overfitting mitigation
Global Average Pooling (global average pooling2d)	Compressed feature representation: 256 units	None, Feature summarization
Flatten (flatten)	Vectorization of features: 256 units	None, Preparing for dense layers
Fully Connected (dense)	High-capacity feature processing: 1,024 units	Count: 263,168, Learning dense features
Dropout (dropout 1)	Feature selection: 1,024 units	None, Overfitting mitigation
Fully Connected (dense 1)	High-capacity feature processing: 1,024 units	Count: 1,049,600, Learning dense features
Dropout (dropout 2)	Feature selection: 1,024 units	None, Overfitting mitigation
Output Layer (dense 2)	Final decision layer: 3 units (class probabilities)	Count: 10,250, Classification decisions
Total parameters: 1,532,198, Comprising Trainable: 1,532,192, Non-trainable: 6		

Table 2 presents the detailed layer-by-layer architecture of the custom-developed CNN model shown in Figure 4, which serves as the backbone of our WL image classification system. The initial batch normalization layer sets the stage for the network by normalizing the input distribution, which is essential for accelerating the training process.

Following this, a precisely designed sequence of 2D convolutional layers with increasing numbers of units is deployed to systematically enhance feature extraction capabilities. The strategic incorporation of max pooling layers systematically reduces the spatial dimensions of the feature maps, significantly reducing computational demands while maintaining the most salient features.

The employment of dropout layers interspersed among the high-capacity, fully connected dense layers functions as a regulatory mechanism to combat overfitting, ensuring that the model generalizes well to new, unseen data. Activation functions such as ReLU introduce nonlinearity, enabling the model to learn complex patterns. Furthermore, batch normalization layers are incorporated to enhance training stability, and dropout is strategically applied to prevent overfitting.

The final output layer, with its SoftMax activation, is calibrated to output three classes, translating the learned patterns into clinically relevant classifications of working length (optimum, over-extended, or under-extended). The proposed CNN architecture has 1.5 million parameters, balancing model complexity and interpretability, a balance paramount in medical image analysis.

By deploying this dual approach, combining the generalized learning capabilities of pre-trained models with the focused precision of a custom-developed architecture, we aim to create a robust system capable of high accuracy and reliability in classifying dental radiographic images.

This methodology not only leverages the strengths of both architectural paradigms but also ensures the models are finely tuned to meet the specific challenges posed by endodontic imaging tasks.

### 2.4 Fine-tuning and training

The fine-tuning and training of the selected and developed CNN models are critical stages in our study, during which we refine the models to ensure optimal performance on dental radiographic images. This phase is thoroughly designed to fine-tune both pre-trained and custom-developed models, enhancing their ability to accurately identify and classify the varied anatomical features and pathologies evident in endodontic imaging.

Fine-tuning is approached by unfreezing the top layers of the pre-trained models, which are the most likely to capture high-level features specific to our dental dataset. The parameters of these layers are then carefully adjusted through continued training on our dataset (49). We modify:

**Learning Rates**: Initially set lower than usual to prevent the loss of previously learned features. The learning rate is progressively increased using a cyclical policy, which helps escape local minima and explore a broader region of the solution space.
**Layer Configurations**: Depending on the specific model architecture, certain layers may be added or removed to better capture the nuances of dental images. For instance, additional convolutional layers may be added to deepen the model's ability to process fine details, or fully connected layers may be adjusted to refine the output towards the specific classes of interest.

Once the models are fine-tuned, they undergo a rigorous training regime using the following advanced techniques to enhance their generalizability and robustness:

**Dropout**: Applied randomly to neurons in the dense layers during training to prevent overfitting by reducing interdependencies among neurons. This method effectively improves the model's ability to generalize from the training data to unseen data.
**Batch Normalization**: Implemented after every convolutional layer to normalize the activations and accelerate the training process. This technique also helps stabilize the neural network by normalizing the input layer by adjusting the mean and variance.

Both dropout and batch normalization are pivotal for managing internal covariate shift, thereby speeding up training and improving the model's performance during inference on new, unseen images.

Training is conducted in batches, with batch size accurately chosen based on the computational capabilities and the specific architecture being trained. Gradient descent algorithms, specifically Adam or Stochastic Gradient Descent (SGD) with momentum, are utilized to update the weights. These optimizers are selected for their efficiency in handling sparse gradients and robustness in diverse training landscape conditions.

Throughout the training process, the model's performance is continuously monitored using a validation set. This not only ensures the model is not overfit with the training data but also provides insights into how it might perform in a real-world clinical setting. Based on the validation results, the training parameters are iteratively refined to further enhance accuracy and reliability.

Table 3 presents the fine-tuned configuration parameters used to train the convolutional neural networks described in this work. It highlights the software environment and tools, such as Python with TensorFlow and Keras, and emphasizes the advanced computational framework employed. The data is divided into training, validation, and test sets at 80%, 10%, and 10%, respectively, facilitating robust model training and evaluation. A learning rate of 0.0001, combined with the Adam optimization algorithm, ensures efficient convergence during training. Regularization is handled via an L2 penalty with a decay rate of 0.001 to prevent overfitting, thereby improving the models’ generalization. The networks are trained for 128 epochs with a minimum batch size of 64, using a categorical cross-entropy loss function, which is optimal for the multi-class classification problems inherent to this study.

**Table 3:** Detailed fine-tuned configuration parameters for CNN model training.

Parameter	Specification
Development language	Python
Neural network libraries	TensorFlow, Keras
Data split (Train/Validate/Test)	80%/10%/10%
Learning rate	0.0001
Optimizer	Adam
Regularization technique	L2 regularization
L2 regularization rate	0.001
Training epochs	128
Batch size	64
Loss computation	Categorical cross-entropy

This comprehensive fine-tuning and training process is essential for optimizing the performance of our CNN models, ensuring they are not only theoretically sound but also practically viable and effective in a clinical environment.

### 2.5 CNN models’ evaluation and classification process

The evaluation process for CNN models assesses their accuracy and reliability to determine their effectiveness and readiness for deployment in clinical settings. The 22 CNN models were comprehensively evaluated using 11 different metrics that are listed in Table 4. The model with the best performance was then plugged into a web-based tool to offer a system with high accuracy, reliability, and practical usability in clinical applications.

**Table 4:** The detailed evaluation metrics and their definitions.

Evaluation metric	Definition
Accuracy	Measures the overall correctness of the model across all classes, giving us a straightforward indication of performance
Precision (Positive Predictive Value)	Indicates the accuracy of positive predictions for each class, crucial for applications where the cost of a false positive is high.
Recall (Sensitivity or True Positive Rate):	Measures the model's ability to detect all positive instances, which is essential in clinical settings where missing a positive case can have profound implications.
F1-score	Harmonizes precision and recall into a single metric, balancing the trade-offs between them and providing a more comprehensive overview of model performance.
Testing time	Indicates the efficiency of the model, ensuring the model can be integrated smoothly to provide output without significant delays.
Specificity (True negative rate)	Measures the proportion of actual negatives that are correctly identified as such, necessary for confirming the absence of a condition.
Negative predictive value (NVP)	Indicates the likelihood that a negative test truly means the absence of a condition, which is valuable in ensuring the reassurance of negative screening results.
False positive rate (FPR)	Indicates the probability of falsely classifying a negative case as positive, critical in areas where false alarms are costly.
False negative rate (FNR)	Represents the probability of falsely classifying a positive case as negative, which is crucial in settings where missing a condition could be detrimental.
False discovery rate (FDR)	Measures the proportion of false positives in all positive predictions, which is essential for understanding the reliability of positive test results.
False omission rate (FOR)	Measures the proportion of false negatives in all negative predictions, relevant in scenarios where negatives carry significant implications.
Misclassification rate (MR)	Measures the overall rate at which the model incorrectly classifies instances, giving an overall error rate of the system.

## 3 Results and analysis

A comprehensive analysis of the outcomes derived from the detailed evaluation of the CNN models developed to classify working lengths in dental radiographs. Key performance metrics, including accuracy, F1-score, precision, and recall, were computed for all examined models, along with testing time to assess operational efficiency. An in-depth detection assessment was conducted on the most accurate custom-developed CNN model, encompassing all relevant evaluation parameters across the three designated WL categories. In terms of precision, the DCNN model achieved the highest score of 97.07%, closely followed by EfficientNetB5 and VGG16, also known for their deep learning efficiency in image recognition tasks. The recall metric, critical for ensuring correct classification, was also highest for the DCNN model at 97.03%. The DCNN model excelled in both accuracy and operational efficiency, achieving a test time of only 0.548546 s. EfficientNetB5, VGG16, EfficientNetB1, VGG19, and EfficientNetB2 also achieved commendable accuracy, with scores above 95%, underscoring the efficacy of advanced CNN architectures in handling complex image data. Table 5 presents the detailed detection assessment parameters for each examined AI model.

**Table 5:** Detection assessment parameters of the examined AI models.

AI model	Accuracy	F1-score	Precision	Recall	Testing time (sec)
DCNN (Scratch)	**0.970297**	**0.970390**	**0.970702**	**0.970297**	**0.548546**
EfficientNetB5	0.960396	0.960613	0.961948	0.960396	6.703499
VGG16	0.957096	0.957219	0.957729	0.957096	7.026967
EfficientNetB1	0.957096	0.957182	0.957879	0.957095	2.888323
VGG19	0.950495	0.950694	0.951500	0.950495	9.019332
EfficientNetB2	0.953795	0.953937	0.954361	0.953795	3.007454
EfficientNetB7	0.947195	0.947434	0.947956	0.947195	12.19628
MobileNetV3Large	0.947195	0.947207	0.948072	0.947195	1.444528
EfficientNetB3	0.940594	0.940899	0.941771	0.940594	3.825811
EfficientNetB6	0.943894	0.943810	0.944299	0.943894	9.057463
ResNet50	0.930693	0.931268	0.933418	0.930693	4.158439
MobileNet	0.927393	0.927840	0.928913	0.927393	1.177610
EfficientNetB0	0.927393	0.927491	0.928669	0.927393	2.072861
DenseNet121	0.927393	0.927351	0.927517	0.927392	4.216988
EfficientNetB4	0.927393	0.927691	0.928396	0.927393	4.960827
MobileNetV3Small	0.920792	0.920765	0.921505	0.920792	0.878270
DenseNet169	0.920792	0.916644	0.922791	0.920792	5.148977
DenseNet201	0.920792	0.920544	0.922299	0.920792	6.699370
Xception	0.910891	0.910857	0.911724	0.910891	3.529581
InceptionV3	0.891089	0.891423	0.892822	0.891089	2.156583
MobileNetV2	0.864686	0.864373	0.868220	0.864686	1.380209
InceptionResNetV2	0.617161	0.614039	0.614189	0.617161	5.616386

The custom-developed deep CNN (DCNN) (Scratch) model achieved superior performance, with an accuracy of 97.02% and an F1-score of 97.03%, indicating highly reliable classification. Table 6 outlines the detection assessment parameters for the DCNN model for the optimum, over-extended, and under-extended WL categories.

**Table 6:** Detailed detection assessment parameters of the most accurate DCNN model across the three examined working length samples.

Metric	Optimum WL	Over-extended WL	Under-extended WL
Acc.	0.970297	0.990099	0.980198
TNR	0.970297	0.995050	0.990099
NPV	0.984925	0.990147	0.980392
FPR	0.029703	0.004950	0.009901
FNR	0.029702	0.019801	0.039604
PPV	0.942307	0.990000	0.979798
FDR	0.057692	0.010000	0.020202
TPR	0.970297	0.980198	0.960396
FOR	0.015075	0.009852	0.019608
F1-score	0.956097	0.985074	0.970000
MR	0.029703	0.009900	0.019802

Additionally, to conduct a deep analysis, we evaluated the performance of the top six CNN models based on their accuracy and loss curves, and the precision of their classification capabilities as depicted in their confusion matrices. These analyses provide insights into the models’ abilities to learn and generalize from the training data, as well as their precision in classifying working lengths.

**Figure 5:**
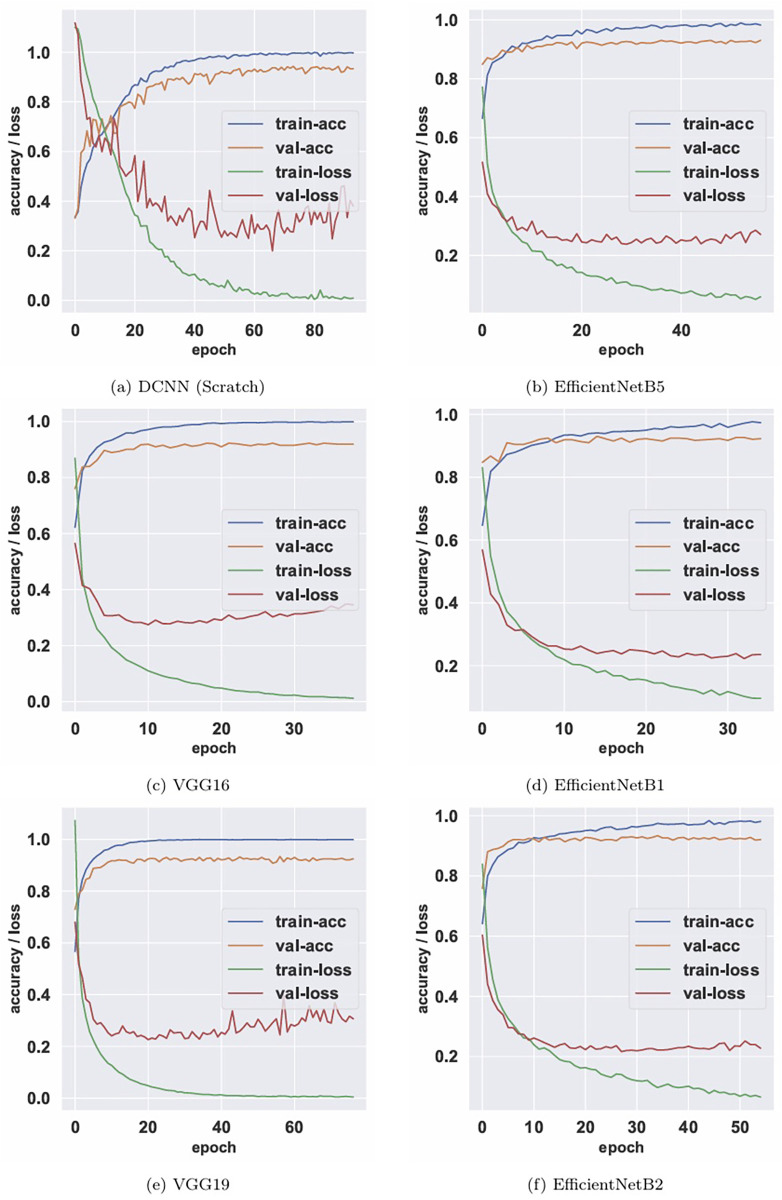
Accuracy and loss curves for the top six most accurate AI models.

The accuracy and loss curves, illustrated in Figure 5 [(a)–(f)], represent key indicators of model performance over training epochs for the top-performing models such as DCNN, EfficientNetB5, VGG16, EfficientNetB1, VGG19, and EfficientNetB2.

In Figure 5(a), a slight increase in the validation loss is observed after approximately epoch 40. This corresponds to the inflection point where the DCNN achieves its optimal generalization. Beyond this stage, further minimization of the training objective can lead to mild overfitting, as the learned feature representations become increasingly tailored to the training data. Moreover, because categorical cross-entropy is highly sensitive to prediction confidence, small variations in probability estimates for challenging validation instances can cause the loss value to rise without a corresponding decrease in classification accuracy. To ensure optimal performance, the system used a model checkpointing approach, saving and loading the best parameters identified before this trend emerged, thereby maintaining the stability and reliability reported in the final evaluation metrics.

Notably, the DCNN model exhibits rapid stabilization in accuracy and a corresponding decrease in loss, achieving both high training accuracy and low validation loss early in training. This suggests an effective learning process with minimal overfitting. EfficientNetB5 and VGG16 also show exemplary convergence behavior, indicating robustness in their learning mechanisms.

**Figure 6:**
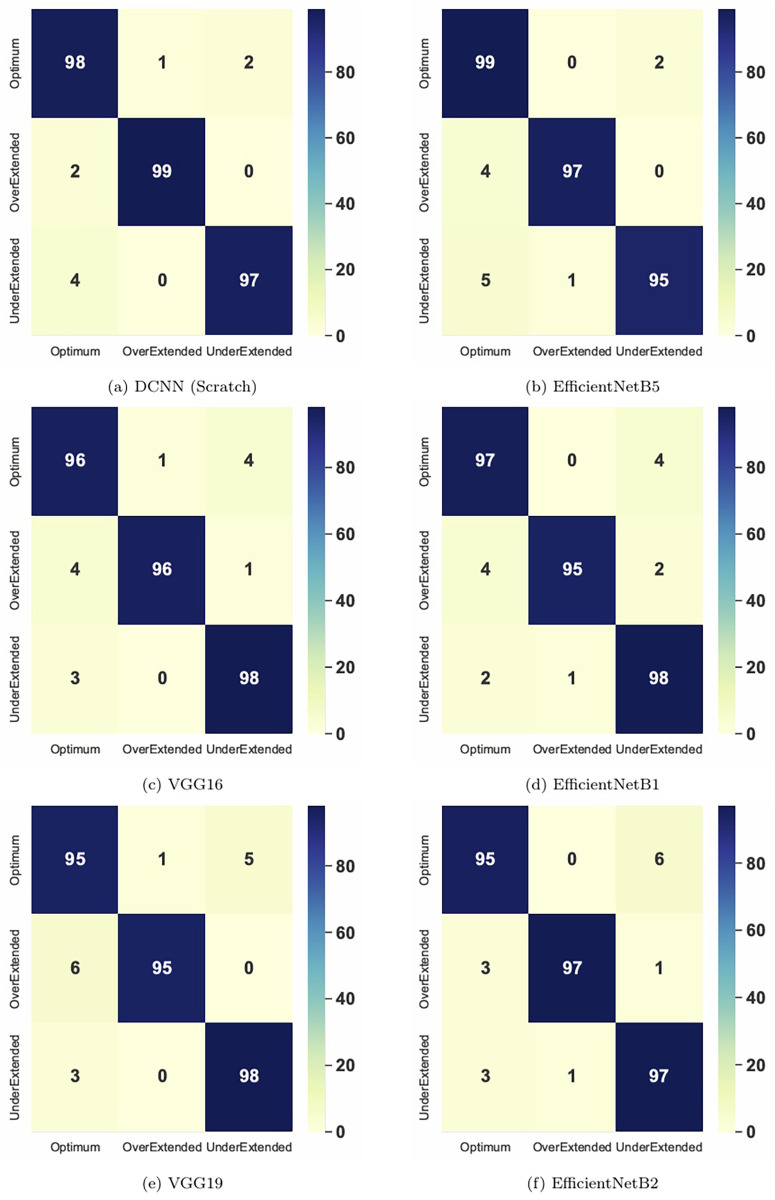
Confusion matrices for the top six most accurate AI models.

Figure 6 [(a)–(f)] displays the confusion matrices for the same subset of top-performing CNN models, offering a breakdown of their predictive performance across the three classified working lengths: optimum, over-extended, and under-extended. The matrices show high true-positive rates across all six CNN models, with the DCNN achieving near-perfect classification across all categories. The EfficientNetB5 model shows a slight misclassification between the optimal and over-extended categories, suggesting opportunities to improve at distinguishing closely related classes.

## 4 Web-based smart WL feedback system: case study

**Figure 7:**
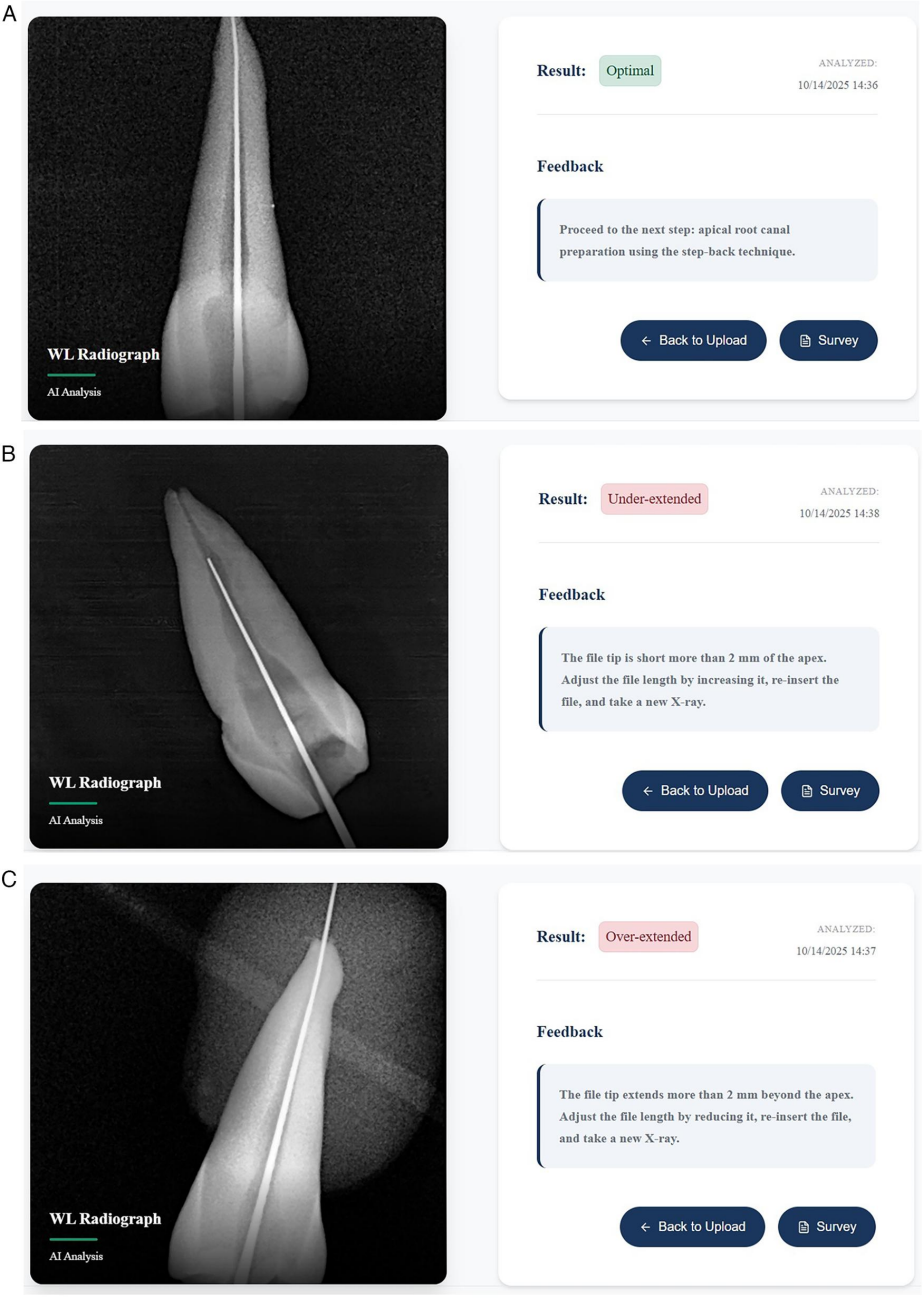
**(A)** case study student's submission of optimal WL radiograph to the AI tool and the feedback received promptly. **(B)** Case study student's submission of underextended WL radiograph to the AI tool and the feedback received promptly. **(C)** Case study student's submission of overextended WL radiograph to the AI tool and the feedback received promptly.

The best AI model was integrated into a web-based service that allows students to submit their work (WL radiograph) and receive prompt, constructive feedback without delay. For instance, consider the three different inputs from three students, as shown in Figure 7. The feedback coming from our automated smart system was as follows:

For (**code 0**), Figure 7A, the system classifies it as an optimal position since the file tip is within 2 millimeters (mm) of the radiographic apex. The student is then instructed to proceed to the next step, which involves chemo-mechanical root canal preparation.
For (**code 1**), Figure 7B, the system classifies it as under-extended since the file tip falls short of the radiographic apex by more than 2 mm. The student is then instructed to adjust the file length by increasing it, re-inserting it, and taking a new radiograph.
For (**code 2**), Figure 7C, the system classifies it as over-extended since the file tip extends beyond the radiographic apex by more than 2 mm. The student is then instructed to adjust the file length by reducing it, re-inserting it, and taking a new radiograph.

Thirty dental students completed the survey evaluating the AI-assisted laboratory feedback tool. These 30 students, representing approximately 10% of the total enrolled pre-clinical cohort, were selected for piloting purposes.

The sampling frame included all pre-clinical students at the end of their laboratory training period. Inclusion criteria were A) completion of the pre-clinical endodontic laboratory course, and B) willingness to test the web-based tool. Exclusion criteria included: A) incomplete laboratory attendance, or B) prior participation in tool development. No compensation or incentives were offered; participation was voluntary.

The overall mean rating was 4.67/5, indicating strong satisfaction. The highest-rated item was ‘*Instructions for using the tool were clear and sufficient*’ (mean = 4.74). The remaining rates for the other items questioned in the survey are listed in Table 7. However, because Likert data are ordinal, the median score across all items was 5.0, with IQR values ranging from 4 to 5 to 5–5.

**Table 7:** Summary of student responses to the AI-assisted feedback tool (*n* = 30).

Qualitative study results analysis		
Statement	Mean ± SD	Interpretation
The tool was easy to set up and use	4.71 ± 0.46	Strongly agree
Instructions for using the tool were clear and sufficient	4.74 ± 0.44	Strongly agree
The tool functioned reliably without technical difficulties	4.68 ± 0.47	Strongly agree
Using the tool allowed me to complete the laboratory tasks efficiently	4.55 ± 0.51	Agree

Internal consistency of the questionnaire was assessed using Cronbach's alpha, which demonstrated excellent reliability (*α* = 0.96).

The questionnaire consisted of 10 items covering usability, clarity of instructions, reliability, feedback quality, and perceived learning enhancement. Items were scored using a five-point Likert scale (1 = strongly disagree to 5 = strongly agree). Although this was a custom instrument tailored to the WL determination context, a standardized metric such as SUS or UMUX-Lite will be considered in future work for broader comparability.

Sixteen female students and fourteen males participated in this survey. The web-based tool is hosted on a web server, and its link was shared with the relevant parties. Students consistently agreed that the tool was easy to use, provided clear and timely feedback, and supported efficient task completion. Overall, the tool was perceived as reliable, user-friendly, and beneficial for enhancing learning in endodontic laboratory training.

## 5 Discussions

In Endodontics, AI is increasingly integrated into the technical assessment of root canal treatment through radiographic examination (19, 26, 36, 50, 51). Stages such as WL determination are critical steps in root canal treatment and directly affect its success (6, 7, 32). Providing personalized feedback to each case during endodontic training can be challenging, but it is essential for enhancing effective learning, especially in large classrooms (33, 52, 53).

This experimental study aimed to significantly enhance the precision and reliability of endodontic pre-clinical training by integrating AI Machine learning models with dental radiography. This proposed model incorporated both pre-trained and custom-developed 22 CNN models. The matrices show high true-positive rates across all six CNN models, with the DCNN achieving near-perfect classification across all categories.

The efficiency of clinical training in dental education, specifically within the context of endodontic training protocols, is fundamentally contingent upon accurately determining working length (WL) 4,6,7. Leveraging advanced CNN capabilities, this study introduces an AI-driven framework that enhances the precision of these measurements.

This precise labeling protocol was critical for training the CNN models, as it encapsulated various scenarios a dental practitioner may encounter, thereby enhancing their accuracy and reliability. A well-balanced and refined dataset of 3,000 samples was created, with care taken to maintain consistent image quality and resolution, ensuring each radiograph was suitable for detailed analysis and machine learning applications. The categorization of radiographic WL as optimal, under-extended, and over-extended WL was considered based on the recommendations for radiographic WL measurements in the literature (6–8). In addition, the distribution of images was balanced across the three categories to avoid dataset bias and enhance the validity of the AI-based learning process.

The DCNN model excelled in both accuracy and operational efficiency, achieving a test time of only 0.5 s. This rapid testing capability is advantageous for real-time applications, enabling the model to be integrated smoothly and providing output without significant delays. This high recall rate is essential in clinical settings where missing or delaying diagnostic information could lead to poor procedural outcomes.

This high accuracy and balanced F1 score suggest that the model effectively manages the trade-off between precision and recall, which is crucial for medical applications where false positives and false negatives carry significant consequences.

The accuracy of the DCNN AI-driven model developed in this study (reaching up to 99%) exceeded the accuracy of other AI-driven models exploring WL determination (ranging from 93% to 96%) (29, 30, 54). This could be justified by the integration of both empirical and advanced AI techniques in this study, in addition to the robust and large dataset used to train this model. The use of a case study for a trial of this model was proven to provide effective feedback to the three main categories of WL radiographs. This indicates that automated, accurate, and quick feedback responses will be provided to students, assisting and supporting instructors in offering constructive feedback during this critical step (33, 55, 56). The AI-based feedback model developed through this work could be employed to deliver instant, precise, and constructive feedback to students. This custom model includes specialized layers and training strategies designed to enhance feature extraction directly pertinent to the nuances of endodontic imaging.

This study represents a laboratory-based exploration of AI and machine learning development, making it one of the first initiatives in this direction. The use of a large, well-organized, and quality-assured dataset of WL radiographic images has contributed to the development of a robust model. The high satisfaction scores indicate that the AI-assisted feedback tool was well accepted by students and effectively supported their learning in pre-clinical endodontic sessions. The tool's clarity, ease of use, and instant feedback appear to enhance both efficiency and confidence during laboratory tasks. Such positive perceptions support the continued integration of AI-driven systems into pre-clinical teaching to promote active and self-directed learning.

The limitations of this study must be acknowledged. Since all radiographs were obtained from a single institution using a single imaging software and device, this can limit generalizability across clinics using different radiographic sensors with varying specs.

Although the model performed well diagnostically, this study did not test whether AI-supported feedback improves learning outcomes. Future controlled research, such as a randomized comparison with standard instruction, is required to determine educational effectiveness. This is an alpha version of the tool, and we are improving it.

## 6 Conclusions

This study presents the development of an AI-powered automated feedback system designed to enhance pre-clinical endodontic training by assisting students in determining radiographic working length (WL). A newly labeled dataset of 3,000 radiographs was used to train 22 convolutional neural network (CNN) models, achieving up to 99% accuracy, with the best-performing model selected for implementation. The system provides instant, constructive feedback to support student learning and technical improvement. Future work will focus on expanding the dataset, testing additional AI architectures, and integrating other stages of root canal treatment into a unified intelligent training platform for dental education.

## Data Availability

The raw data supporting the conclusions of this article will be made available by the authors, without undue reservation.
